# The Ethical Significance of Post-Vaccination COVID-19 Transmission Dynamics

**DOI:** 10.1007/s11673-022-10223-6

**Published:** 2022-12-21

**Authors:** Steven R. Kraaijeveld

**Affiliations:** Wageningen University & Research, Wageningen, The Netherlands

**Keywords:** Vaccination policy, Public health ethics, COVID-19 vaccination, Coercion, Vaccine passports, Mandatory vaccination

## Abstract

The potential for vaccines to prevent the spread of infectious diseases is crucial for vaccination policy and ethics. In this paper, I discuss recent evidence that the current COVID-19 vaccines have only a modest and short-lived effect on reducing SARS-CoV-2 transmission and argue that this has at least four important ethical implications. First, getting vaccinated against COVID-19 should be seen primarily as a self-protective choice for individuals. Second, moral condemnation of unvaccinated people for causing direct harm to others is unjustified. Third, the case for a harm-based moral obligation to get vaccinated against COVID-19 is weak. Finally, and perhaps most significantly, coercive COVID-19 vaccination policies (e.g., measures that exclude unvaccinated people from society) cannot be directly justified by the harm principle.

## Introduction 

Vaccines represent a hugely important development in public health (Feemster 2018), not only because they can prevent people from becoming ill but also because vaccines can often stop an individual from spreading disease to others (Orenstein and Ahmed 2017; Verweij 2005). Vaccine developers ideally aim for sterilizing immunity, which is a long-term immune response that can “rapidly prevent a returning virus from gaining ground in the body,” although not all vaccines or infections will produce the necessary neutralizing antibodies (Ledford 2020, 21). While some vaccines only protect individual recipients (e.g., against tetanus), other vaccines can also have beneficial health effects for people beyond individual recipients, which is an important factor when it comes to the ethics of vaccination (Kraaijeveld 2020a). When a vaccine provides sterilizing immunity, the strongest case can be made that, by getting vaccinated, individuals thereby also protect others. After all, if one can no longer transmit a disease post-vaccination, then one is prevented from spreading that disease—and the harms associated with it—to other people.

There were early hopes and signs that the current vaccines against the novel coronavirus disease (COVID-19) would be able to provide sterilizing immunity to SARS-CoV-2, which would diminish the risk of people with minimal symptoms spreading the virus widely (Ledford 2020). Unfortunately, we now know that the vaccines have not been able to confer sterilizing immunity (Vashishtha and Kumar 2022). This does not necessarily mean that the vaccines do not affect transmission rates, but it does mean that a more sophisticated understanding of COVID-19 transmission dynamics is necessary to evaluate the ethics of certain vaccination policies. This is especially true when the policies are restrictive of individual liberties and when they are coercive in nature. While there are different definitions of coercion, I adopt the general view that it is “a condition in which someone is forced to do X, for example, vaccinating one’s children, in the sense that she is left with ‘no reasonable choice’ or ‘no acceptable alternative’ […] but to do X when she would otherwise not choose to do X” (Giubilini 2019, 68).

Coercion may be used in public health, but it requires strong ethical justification (Biglan 2015). According to the harm principle, originally formulated by John Stuart Mill, governments may justifiably coerce citizens or curtail their freedoms only in order to prevent harm to third parties (Mill 2005/1859). For Mill, preventing harm to others is a necessary—if not a sufficient—condition for states to limit individual freedoms. The harm principle is a central tenet in public health ethics that can provide an ethical justification for coercive public health measures generally (Holland 2015), and for coercive infectious disease measures and vaccination policies more specifically (Krom 2011; Amin et al. 2012). Yet, even when the harm principle should apply, this does not automatically justify coercive policies. It is a necessary but not a sufficient condition for coercion. Other principles, like proportionality and subsidiarity, must also be taken into account, so that coercive public health measures are justified “only if they are the sole, or incontestably the most effective, way to achieve [an] outcome, and if the benefits associated with this outcome outweigh the social damage thereby produced” (Haire et al. 2018).

In many countries around the world, coercive vaccination policies have either already been implemented or are still being considered as a means to increase COVID-19 vaccine uptake. Preventing people from being able to work, to make use of public transportation, or to attend college unless they are vaccinated are examples of coercive measures (in light of the above definition) that have been adopted in many places around the world (e.g., Giuffrida 2022; Bardosh et al. 2022b). In Italy, compulsory vaccination for people over the age of 50 has even been implemented (Giuffrida 2022), and there are serious discussions about compulsory vaccination for the general population in other countries, like Austria (Chadwick 2022). The harm principle is often provided as a justification for these coercive measures. It is, presumably, the rationale for measures like COVID-19 vaccine passports and vaccine mandates. If such measures did not prevent harm to third parties, it is unclear what their public health justification would be—even if coercive policies were morally neutral and even if they never led to collateral harms (which, as I will argue later, is not the case).

In principle, then, the harm principle could ethically justify coercive COVID-19 vaccine measures. Whether or not it actually does, however, critically depends on whether the current vaccines substantially prevent people from spreading infection and thereby harming others. Sterilizing immunity might not be necessary, but there must be a significant post-vaccination reduction in SARS-CoV-2 transmission rates for coercive mandates to be directly justified by the harm principle. Should it turn out that the COVID-19 vaccines do not substantially reduce transmission, then the harm principle cannot directly justify coercive vaccination policies.

In this paper, I discuss evidence that the effects of current COVID-19 vaccines on transmission are modest and temporary at best. I argue that this has at least four ethical implications. First, getting vaccinated against COVID-19 should primarily be seen as a self-protective choice for individuals. Second, moral condemnation of unvaccinated people for causing direct harm to others is unjustified. Third, the case for a harm-based moral obligation to get vaccinated against COVID-19 is weak. Finally, and perhaps most importantly, coercive vaccination policies (e.g., those that exclude unvaccinated people from society) cannot be directly justified by the harm principle.

## Post-Vaccination COVID-19 Transmission

By now, there are numerous examples of “breakthrough” infections among groups of fully vaccinated people (Steinbuch 2022; Quiroz-Gutierrez 2022). More systematic and controlled studies are, of course, needed to determine the extent to which vaccines might nevertheless reduce SARS-CoV-2 transmission.

Early evidence that the vaccines significantly reduced SARS-CoV-2 transmission was provided by a study published in the *New England Journal of Medicine*. It found that, in households of vaccinated people, the likelihood of household transmission was approximately 40 to 50 per cent lower than in households of unvaccinated people (Harris et al. 2021). This finding was widely publicized and is still sometimes used as evidence that vaccines substantially reduce, if not prevent, transmission (e.g., U.K. Health Security Agency (2022a). I will refer to this study again later, but for now it should be noted that the data were gathered between January 4 and February 28 of 2021. Since then, the epidemiological characteristics of the pandemic appear to have changed, as is suggested by the following evidence.

A later study published in *The Lancet Infectious Diseases* investigated differences in transmission dynamics between vaccinated and unvaccinated individuals. More specifically, it explored the difference in infection risks of household transmission and found that the secondary attack rates (SAR) among household contacts exposed to vaccinated or unvaccinated people was, respectively, 25 per cent and 23 per cent (Singanayagam et al. 2021). Based on this study, the conclusion may be drawn that the effect of the vaccine on reducing transmission is minimal (Wilder-Smith 2021). The study furthermore examined transmission and viral load kinetics in vaccinated and unvaccinated individuals with mild Delta infection. It found that fully vaccinated individuals with breakthrough infections “have peak viral load similar to unvaccinated cases and can efficiently transmit infection in household settings, including to fully vaccinated contacts” (Singanayagam et al. 2021). If vaccinated and unvaccinated people do not significantly differ in peak viral load when infected, there is little reason to assume that infectivity would nonetheless significantly differ between the groups. These findings are additionally supported by a study that found no significant difference in cycle threshold values between vaccinated and unvaccinated individuals—both asymptomatic and asymptomatic—infected with Delta (Acharya et al. 2021).

Another study examined the relationship between the percentage of populations fully vaccinated and new COVID-19 cases across sixty-eight countries and 2,947 U.S. counties. It found no significant signal of COVID-19 cases decreasing with a higher percentage of populations fully vaccinated; at a country level, the trendline even suggested a marginally positive association between a higher percentage of populations fully vaccinated and a higher number of COVID-19 cases per one million people (Subramanian and Kumar 2021).^1^ Replications of studies like this are clearly needed. Yet, infection rate data from highly vaccinated countries also suggest that vaccines do not significantly reduce infection rates among the fully vaccinated. In Denmark, for instance, unvaccinated people infected with Omicron make up only 8.5 per cent of the total number of infections (Statens Serum Institute 2021). In the United Kingdom, among people over the age of thirty, those who are fully vaccinated currently have significantly higher infection rates than those who are unvaccinated (UK Health Security Agency 2022b). Respectively, 81 per cent and 72 per cent of the populations in Denmark and the United Kingdom are fully vaccinated, with 58 per cent and 55 per cent having received an additional dose (Holder 2022). It does not stand to reason that unvaccinated people are major drivers of transmission in these countries; the data do not support this interpretation. If peak viral load is similar between the groups, as several studies have shown, then one would actually expect that in highly vaccinated populations, infections are increasingly occurring among fully vaccinated people (i.e., the relatively larger group).

A study that estimated the number needed to exclude (NNE) for vaccine passports provides additional evidence that unvaccinated people are not the major drivers of transmission. The study found that at least a thousand unvaccinated people must likely be excluded in order to prevent a single SARS-CoV-2 transmission (Prosser, Helfer, and Steiner 2021). If unvaccinated people were disproportionately spreading infection, one would expect the NNE to be much smaller. The authors of the study conclude that excluding unvaccinated people has negligible benefits for reducing transmission in society (Prosser, Helfer, and Steiner 2021).

A recent summary of the evidence regarding post-vaccination transmission in the *BMJ* suggests that, while the vaccines are good at preventing serious infection and hospitalization, the fact that they are “less good at preventing transmission makes policymaking difficult” (Stokel-Walker 2022). Another summary in the *New England Journal of Medicine* characterizes the situation in the following way, namely that “currently available vaccines have only modest effectiveness against mild infection and transmission, which is further reduced in the context of the newly emerging omicron subvariants” (Nohynek and Wilder-Smith 2022). While post-vaccination transmission rates were found to be lower compared to transmission rates for unvaccinated people without previous infection, they were not found to be significantly lower compared to rates for previously infected unvaccinated people—which is likely to be the majority of unvaccinated people by now—and the post-vaccination effect on transmission generally “doesn't last for long” (Scully 2022).^2^

What might be responsible for the minimal effect of COVID-19 vaccines on transmission? The science does not appear to be settled yet on this question, which in any case cannot be addressed here. One reason may be related to the prevalence of and specific characteristics of Omicron, which is now dominant in many countries. A recent study from Israel examining the effectiveness of a widely administered fourth dose showed a “slightly higher” increase in antibodies than the third dose, but “the increased antibodies did not prevent the spread of infection” (Federman 2022). Early estimates of reduced transmission may perhaps have held when Delta circulated widely (as in the study by Harris et al. [2021]), but they no longer seem to hold with Omicron. A potential 40 to 50 per cent reduction in post-vaccination transmission no longer seems realistic—it is contradicted by more recent studies and by the Omicron infection rates among the fully vaccinated in many countries around the world.

In sum, it must be concluded at this point that the current vaccines have only a modest and transient effect on reducing SARS-COV-2 transmission. What this means for vaccination policy is a pressing and ongoing question. In what follows, I explore some of the ethical implications.

## Ethical Implications

If COVID-19 vaccines have only a relatively small and short-lived effect on transmission, this gives rise to at least four important ethical implications.^3^

First, because the vaccines still significantly reduce the personal risk of COVID-19-related hospitalization and death (Zheng et al. 2022), getting vaccinated against COVID-19 should be considered primarily as a self-protective choice from the perspective of individuals (cf. Kraaijeveld 2020a). The most compelling reason for a person to get vaccinated against COVID-19, in other words, is to protect oneself. From the perspective of governments, COVID-19 vaccination might be said to be chiefly a paternalistic intervention; although a more indirect version of the harm principle may still be relevant, for instance when vaccination choices put pressure on healthcare systems.^4^ It must be noted, however, that (1) for many potentially and even likely self-injurious activities (e.g., extreme sports) through which people risk needing healthcare services, we do not generally accept coercive interventions, and (2) coercive measures that appeal to healthcare pressures apply only when such pressures exist, which does not seem to offer a stable basis for long-term health policy. Furthermore, given that health is a basic human right that creates a legal obligation on states “to ensure access to timely, acceptable, and affordable health care of appropriate quality” (World Health Organization 2017), and given that access to healthcare itself is arguably a human right (Denier 2005), states cannot indefinitely place responsibility for healthcare (e.g., by appealing to systematic pressures) on individual citizens without also taking responsibility themselves (e.g., by increasing healthcare capacity, supporting healthcare workers, etc.).

Second, given that there is support neither for the judgment that by not getting vaccinated a person is thereby directly harming others, nor for the corollary that by getting vaccinated a person thereby directly avoids harming others, the moralization of vaccination status—and especially the moral condemnation and social exclusion of unvaccinated people—is unjustified on those grounds. There have already been appeals to stop publicly discriminating against unvaccinated people (e.g., by Amnesty International [Piovaccari 2022]). Kraaijeveld and Jamrozik (2022) have recently introduced and developed the concept of mismoralization, which is when moralization is morally inappropriate. They argue that moralization of COVID-19 vaccination status constitutes a case of mismoralization in public heath, given that it is unjustified from a metaethical perspective. Given the potential negative effects of widespread moralization (e.g., stigmatization, dehumanization, ostracism, social conflict, etc.),^5^ it is imperative that it be addressed and ameliorated wherever possible—both for the sake of potentially affected individuals, as well as for the general functioning and well-being of society. Whatever might be objectionable about people’s decisions not to get vaccinated against COVID-19, unvaccinated people cannot justifiably be blamed, condemned, or ostracized for directly causing harm to others.

Third, given that the link between not getting vaccinated and directly harming others is tenuous at best, the case for a moral obligation to get vaccinated is weak to the extent that such an obligation would be grounded in the obligation to avoid harm to others (Ivanković and Savić 2021). If harm to others cannot concretely be averted by getting vaccinated, then it difficult to see why one should nevertheless have a moral obligation to get vaccinated based explicitly on a duty to avoid harm to others. Individual moral obligation to get vaccinated, or COVID-19 vaccine mandates more generally, may still be grounded in other principles, like solidarity (Yeh 2022)^6^ or fair contribution to herd immunity as a public good (e.g., Giubilini, Douglas, and Savulescu 2018). Given that I am specifically concerned in this paper with harm and harm prevention, I will not address other approaches here. It should be noted, however, that fairness-based approaches often presuppose that vaccine-induced herd immunity (i.e., the public good in question) is a possibility, which scientists are increasingly considering to be impossible in the case of COVID-19 (Aschwanden 2021; Bruemmer 2022).

Finally, the modest and temporary effects of COVID-19 vaccines on transmission means that the harm principle in itself cannot justify coercive vaccination policies. The difference in the propensity to cause harm to others between vaccinated and unvaccinated people is insufficiently substantial for the harm principle to hold directly. Clear ethical grounds are needed for governments to be justified in taking highly coercive measures to steer people toward getting vaccinated against COVID-19. As Verweij and Dawson have argued, participation in vaccination programmes “should, generally, be voluntary because of the importance now given to autonomous decision making by competent adults in health care” (2004, 3125). In some cases, as I have suggested earlier, the harm principle could provide an ethical justification for coercive vaccination policies—but there has to be a real and a reasonable sense in which they stop one party from harming others or more generally “prevent a concrete and serious harm” (Verweij and Dawson 2004, 3123). What we currently know about COVID-19 post-vaccination transmission dynamics does not provide a concrete harm-prevention ground for coercion. Yet, public health interventions, even in times of uncertainty, must be ethically defensible and communicable to the public (Ho and Huang 2021). Public health officials must be able to explain why the minimal effects of the vaccines on transmission nonetheless warrant coercive vaccine mandates—especially in light of the many small risks of harm to others that we permissibly take in other ways and in different areas of life (cf. Hansson 2003).

The idea that one avoids harming others by getting vaccinated is pervasive and, if untrue, also potentially deleterious. If people mistakenly believe that getting vaccinated against COVID-19 will protect others, then they may alter their behaviour accordingly—and, paradoxically, increase their risk of infecting others. This is a real concern, for many influential public health communications still urge people to get vaccinated, “To Protect Yourself, Your Coworkers, Your Patients, Your Family, and Your Community” (United Nations 2022). Furthermore, should people learn that the case for protecting others by getting vaccinated is not as strong as public health officials have communicated it to be, then this could lead to reactance and a larger breakdown of public trust and support for COVID-19 measures. The many stories in the media about infections in groups of fully vaccinated people have already cast public doubt on the idea that the vaccines are preventing transmission.

My argument, then, is that in light of the most recent evidence regarding post-vaccination COVID-19 transmission, the harm principle does not provide a direct justification for coercive vaccination policies (e.g., those that would exclude unvaccinated people from public spaces). Some have argued that mandatory vaccination can never be justified (Kowalik 2021). I do not necessarily argue that here. Mandates might be justified; for instance, for individuals who are at highest risk of severe illness from COVID-19 (Williams 2021). But as a basis for sustainable and far-sighted COVID-19 public health policy, coercion should be (re)considered very carefully. In the long run, coercion is often counterproductive. Coercive measures can seriously undermine trust, which is an invaluable resource in healthcare and for the longevity of vaccination policies (Gur-Arie, Jamrozik, and Kingori 2021a). If the public health goal is to increase vaccine uptake, coercive measures can actually increase hesitancy and ultimately decrease uptake (Bester 2015; Haire et al. 2018). Persuasion may ultimately be a better means of promoting COVID-19 vaccination than coercion or incentivization (Pennings and Symons 2021).^7^ Research suggests that people generally respond to the idea of getting vaccinated for the sake of others (Böhm and Betsch 2022; Kraaijeveld and Mulder 2022); some might respond to altruistic reasons (i.e., reasons beyond self-protection) even if the effects of the COVID-19 vaccines on transmission are minimal. There are important moral reasons for governments to leave room for citizens to be able to engage in altruistic behaviour, especially in difficult times (Kraaijeveld 2020b). Finally, coercive measures may in and of themselves sometimes cause harm. According to Bardosh and colleagues, the COVID-19 vaccine mandates, passports, and restrictions that have been widely adopted around the world may be causing more harm than good. Their comprehensive analysis “strongly suggests that mandatory COVID-19 vaccine policies have had damaging effects on public trust, vaccine confidence, political polarization, human rights, inequities, and social wellbeing” (Bardosh et al. 2022a, 1). These potential harms are clearly an important consideration in any ethical analysis of coercive public health measures. Healthcare unions in the United Kingdom have recently voiced concerns that mandatory COVID-19 vaccination for healthcare workers risks worsening the current staffing crisis and threatens to undermine healthcare provision at a time of great pressure and need (Waters 2022). Given that many healthcare workers have already been infected with SARS-CoV-2 (Gholami et al. 2021), and given that previous infection has been found to offer robust protection that can last for at least thirteen months (Kojima and Klausner 2022; Kim et al. 2021), there does not seem to be a strong ethical case for mandates that target healthcare workers—especially in light of the minimal effects of the current vaccines on reducing transmission.^8^ Differentiated measures for unvaccinated individuals require clear goals and strong justifications (Voo et al. 2022), and others have argued that  policy-makers should in any case not discriminate against natural or post-infection immunity when it comes to vaccine mandates (Pugh et al. 2022; Tan et al. 2022).

In conclusion, the latest evidence that the current COVID-19 vaccines have only a modest and transient effect on transmission raises important ethical questions. Perhaps most pressing for vaccination policy is that the harm principle does not appear to provide substantial grounds for coercive vaccination policies like mandates, passports, and other restrictions. Early on in the pandemic, there was a call for public health agencies and governments to “do better in transparently communicating […] the justifications for restrictive interventions, and the long-term all-things-considered goals of public health policy” (Jamrozik and Heriot 2020, 1169). Some two years later at the time of writing, when vaccine mandates of unprecedented scope and scale have already been introduced or are on the horizon, I echo this call. Transparency about the ethical justification of coercive and exclusionary COVID-19 vaccination policies is all the more urgent.

## Footnotes

1 It should be noted that the original study has been criticized by Backhaus (2021).

2 It should be noted that these findings are based on a preprint article (Sophia et al. 2022).

3 There is another potential consequence, namely for the ethics of vaccinating children against COVID-19 for the sake of others, which I will not address in this paper (for discussions of the significance of the modest effects of COVID-19 vaccines on transmission in this area, see Giubilini 2021; Kraaijeveld, Gur-Arie, and Jamrozik 2022; Gur-Arie, Kraaijeveld, and Jamrozik 2021b).

4 To the extent that vaccination reduces harms for individuals, there may also be indirect effects on other people, for instance people who depend on those individuals (e.g., children). My concern is with the direct application of the harm principle, so that, while these potential collateral harms are certainly important to consider, they do not directly affect my arguments regarding coercive policies based on the harm principle. It should also be noted that the vaccines offer imperfect protection, which further complicates discussions about these kinds of collateral harms. Thanks to an anonymous reviewer for raising this issue.

5 For an overview of the negative effects of moralization in public health, and for potential ways to address inappropriate moralization, see Kraaijeveld and Jamrozik (2022).

6 See Barbara Prainsack (2022) for persuasive criticism of COVID-19 mandates grounded in the concept of solidarity.

7 While I have argued that coercive vaccination policies cannot be directly justified by the harm principle, this should not be taken to imply that governments cannot or should not employ other (i.e., non-coercive) measures (e.g., persuasion, information campaigns, etc.) in order to encourage vaccination, for instance as proposed by Pennings and Symons (2021).

8 In general, it seems that there is a stronger *prima facie* reason for vaccine mandates for healthcare workers than for the general public, given that the former group is more likely to be in close contact with vulnerable people. Whether such mandates are justified *all things considered* is, of course, subject to a number of additional considerations. On the other hand, if mandates for healthcare workers are not ethically justified, it is difficult to see on what grounds mandates for the general public would be justified.
